# Serdemetan Antagonizes the Mdm2-HIF1α Axis Leading to Decreased Levels of Glycolytic Enzymes

**DOI:** 10.1371/journal.pone.0074741

**Published:** 2013-09-06

**Authors:** Jason A. Lehman, Paula M. Hauck, Jaimie M. Gendron, Christopher N. Batuello, Jacob A. Eitel, Allan Albig, Madhavi P. Kadakia, Lindsey D. Mayo

**Affiliations:** 1 Department of Pediatrics, Herman B Wells Center for Pediatrics Research, Indianapolis, Indiana, United States of America; 2 Department of Biochemistry and Molecular Biology, Indiana University School of Medicine, Indianapolis, Indiana, United States of America; 3 Department of Biology; Boise State University, Boise, Idaho, United States of America; 4 Department of Biochemistry and Molecular Biology, Boonshoft School of Medicine, Wright State University, Dayton, Ohio, United States of America; 5 Indiana University Simon Cancer Center, Indiana University School of Medicine, Indianapolis, Indiana, United States of America; Rush University Medical Center, United States of America

## Abstract

Serdemetan (JNJ-26854165), an antagonist to Mdm2, was anticipated to promote the activation of p53. While regulation of p53 by Mdm2 is important, Mdm2 also regulates numerous proteins involved in diverse cellular functions. We investigated if Serdemetan would alter the Mdm2-HIF1α axis and affect cell survival in human glioblastoma cells independently of p53. Treatment of cells with Serdemetan under hypoxia resulted in a decrease in HIF1α levels. HIF1α downstream targets, VEGF and the glycolytic enzymes (enolase, phosphoglycerate kinase1/2, and glucose transporter 1), were all decreased in response to Serdemetan. The involvement of Mdm2 in regulating gene expression of glycolytic enzymes raises the possibility of side effects associated with therapeutically targeting Mdm2.

## Introduction

Mdm2 is part of an ubiquitin ligase complex and is commonly known to target p53 tumor suppressor protein for ubiquitination. p53 is frequently lost or mutated in human cancers, while Mdm2 is found to be highly overexpressed in multiple types of cancer. These alterations in Mdm2 and p53 levels contribute to the refractory nature of cancer cells to initiate apoptosis. While the pathways whereby Mdm2 can provide resistance to apoptosis are not well understood, one possible mechanism is the complex formation of Mdm2 and Hypoxia inducible factor 1α (HIF1α), a transcription factor activated in response to hypoxic stimuli. HIF1α promotes angiogenesis and upregulates metabolic genes, which are necessary to sustain tumor cells [1], [2]. Complex formation with Mdm2 is important for HIF1α stabilization and the induction of vascular endothelial growth factor (VEGF) [3]–[5].

Serdemetan (JNJ-26854165) is a novel small molecule identified by Johnson & Johnson Pharmaceutical R&D as an antagonist to Mdm2. Serdemetan, a tryptamine derivative, can synergize with DNA damaging compounds and elicit a p53 apoptotic response in leukemia cells [6]. In solid tumor cell lines, Serdemetan was observed to enhance radiosensitization and delayed tumor growth by inhibiting proliferation and also blocking the migration of endothelial cells [7]. Recruitment of these endothelial cells by the secretion of factors such as VEGF is necessary for angiogenesis. Angiogenesis and glycolysis are essential for tumor cell survival.

In this study, we examined the affects of hypoxia and Serdemetan on human glioblastoma cell lines that have functional (U87 and SF767) and non-functional p53 (U373). We found that Serdemetan altered the ability of Mdm2 to stabilize HIF1α, which resulted in a decrease in VEGF and other HIF1α targets involved in glycolysis. The decrease in HIF1α levels and downstream targets was evident in glioblastoma cells regardless of p53 status. Moreover, our data provide a novel mechanism whereby the Mdm2-HIF1α axis is responsible for inducing glycolytic genes. Additionally, the survival of all three glioblastoma cell lines was diminished with Serdemetan under hypoxia, implicating a role for Mdm2 in regulating pathways aside from the ascribed function in ablating p53 activity.

## Materials and Methods

### Materials

A working stock concentration of 10 mM JNJ-26854165 (Johnson & Johnson) (Serdemetan) was subsequently diluted to the concentrations given. The antibodies used for detection were: enolase (5A4), GAPDH (6C5), Glut1 (H-43), HIF1α (HIa-67), Mdm2 (SMP14), p21 (C-19), p53 (DO-1), PGK1/2 (A-5), a-tubulin (TU-02), and VEGF (147) from Santa Cruz Biotech. Mdm2 (2A10), Mdm2 (4B11) were obtained from EMD.

### Cell Culture

The human glioblastoma cell lines U87MG, SF767, and U373 (ATCC) were cultured at 37°C in a humidified incubator with 5% CO_2_. All cell lines were maintained in Dulbecco’s modified Eagle’s medium with high glucose (Invitrogen) supplemented with 10% fetal bovine serum and 50 units/mL of penicillin and 50 µg/mL of streptomycin sulfate (Invitrogen). Survival assays were completed by plating 12 well plates with 150,000 cells per well with Serdemetan or DMSO in hypoxia for 48 h. Cells were stained with methylene blue and the dye was liberated using 0.5 mol/L HCl for quantitation by measuring absorbance at 595 nm.

### Western Blotting and Cytoplasmic/nuclear Fractionation

Cells for whole cell lysates were solubilized in lysis buffer: 25 mM Tris HCl, pH 8.0, 150 mM NaCl, 1 mM EDTA, 1 mM EGTA, 1% IGEPAL, 1 mM phenylmethylsulfonyl fluoride (PMSF), 10 µg/mL aprotinin, 10 µg/mL leupeptin, 1 mM sodium orthovanadate (Na_3_VO_4_) and 10 mM sodium fluoride (NaF). Lysates were boiled in 1X Laemmli buffer prior to Western blotting analysis. Nuclear and cytoplasmic extracts were made as previously described [8].

### Reporter Assay

Reporter assay methods have been previously described [4].

## Results

**Figure 1:**
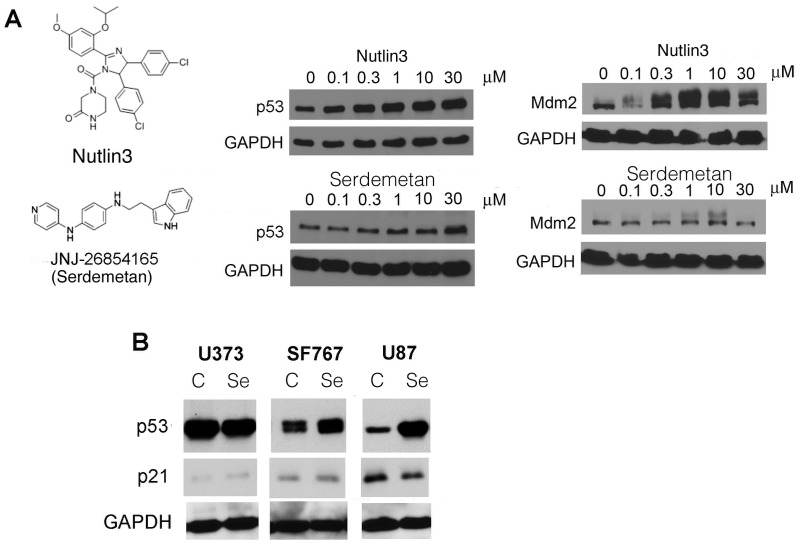
The effect of Serdemetan on p53 levels. **A)** Chemical structure of Serdemetan (SE) and Nutlin3. Western blot of U87 cell lysates treated with increasing concentrations of Nutlin3 or Serdemetan for 6 h to detect p53, Mdm2 and GAPDH. **B)** Western blot of p53, p21 and GAPDH from U373, SF767, and U87. Whole cell lysates were prepared from cells treated with either DMSO or 10 µM Serdemetan (SE) for 6 h.

The p53-Mdm2 interaction was first successfully targeted for pharmacological inhibition using the Nutlin3 compound. Other compounds have been developed to target Mdm2 including Serdemetan (JNJ-26854165). Since Nutlin3 elevates p53 levels through inhibition of Mdm2–p53 binding, we tested whether Serdemetan has a similar mechanism of action. Although Serdemetan led to a dose dependent increase in p53 levels in U87MG cells which plateaued at 30 µM, it did not lead to a robust induction of Mdm2 as seen with Nutlin3. (Fig. 1A). We next examined the effect of Serdemetan on the induction of p21, a downstream target gene of p53, in cells that maintain wild type p53 (U87MG and SF767) or gain of function mutant p53 (U373). Despite the elevated p53 levels, there were no changes in p21 levels (Fig. 1B). These data suggest that p53 is not transcriptionally active with Serdemetan alone, contrary to the reported functions of nutlin3 that cause the induction of p21 [9].

**Figure 2:**
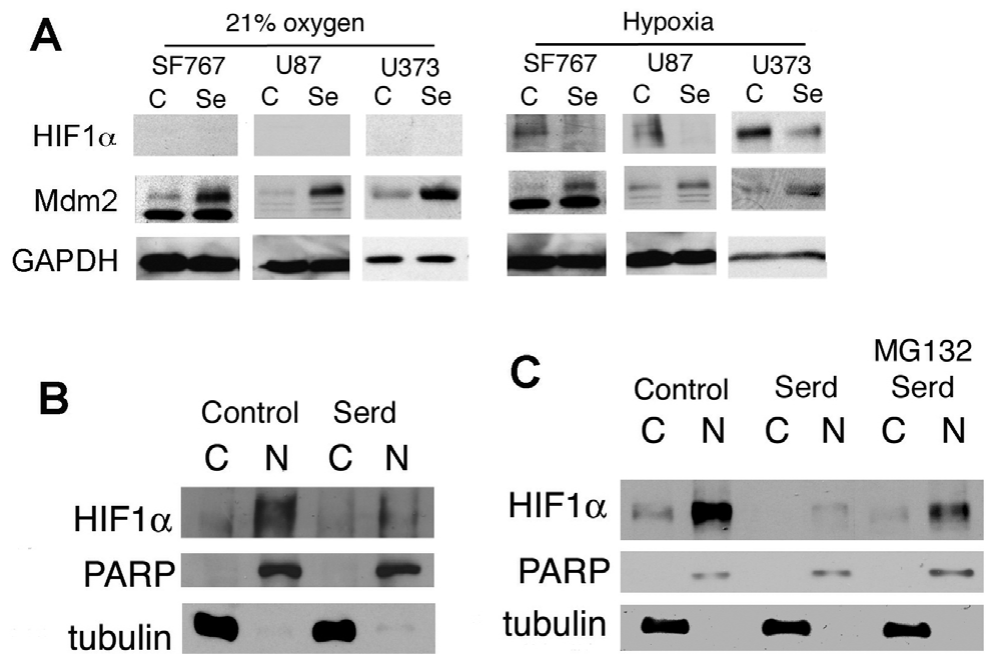
Detection of HIF1α in response to Serdemetan treatment. **A)** Western blot of HIF1α, Mdm2 and GAPDH from SF767, U87 and U373 cellular extracts. Cells were subjected to treatment of either 21% oxygen or hypoxic (1%) conditions for 6 h with 10 µM Serdemetan (Se) or DMSO (C). **B)** Western blot analysis of HIF1α, tubulin, and PARP from cytoplasmic (C) and nuclear (N) extracts of SF767 cells treated with 10 µM of Serdemetan (Serd) or DMSO (Control). **C)** Western blotting was performed for HIF1α, tubulin, and PARP as described above with the addition of pretreatment with 10 µM MG132 for 16 h.

We and others have previously reported that Mdm2 can increase the stability of hypoxia inducible factor 1 alpha (HIF1α) [4], [5]. HIF1α levels and the induction of the downstream target genes, VEGF and EPO, were dramatically decreased when Mdm2 was absent or cells were treated with Nutlin3 [4], [10]. To examine if Serdemetan functions similarly to Nutlin3 on the Mdm2 -HIF1α interplay (4), we analyzed HIF1α levels in the presence of Serdemetan under normoxic and hypoxic conditions. Our data show that HIF1α levels were increased with hypoxia as expected, yet this elevation was not evident in the presence of Serdemetan (Fig. 2A). Upon further examination of nuclear and cytoplasmic extracts from U87 cells treated with Serdemetan, we found that the levels of HIF1α in the nuclear fraction were negligible compared to the DMSO control (Fig. 2B). To determine if the effects of Serdemetan on HIF1α were dependent on the proteasome, pre-treatment with the proteasome inhibitor, MG132, and Serdemetan under hypoxic conditions resulted in detectible levels of HIF1α in the nuclear fraction (Fig. 2C). Together, these results demonstrate that Serdemetan inhibits the stabilizing actions of Mdm2 on HIF1α in hypoxic conditions.

**Figure 3:**
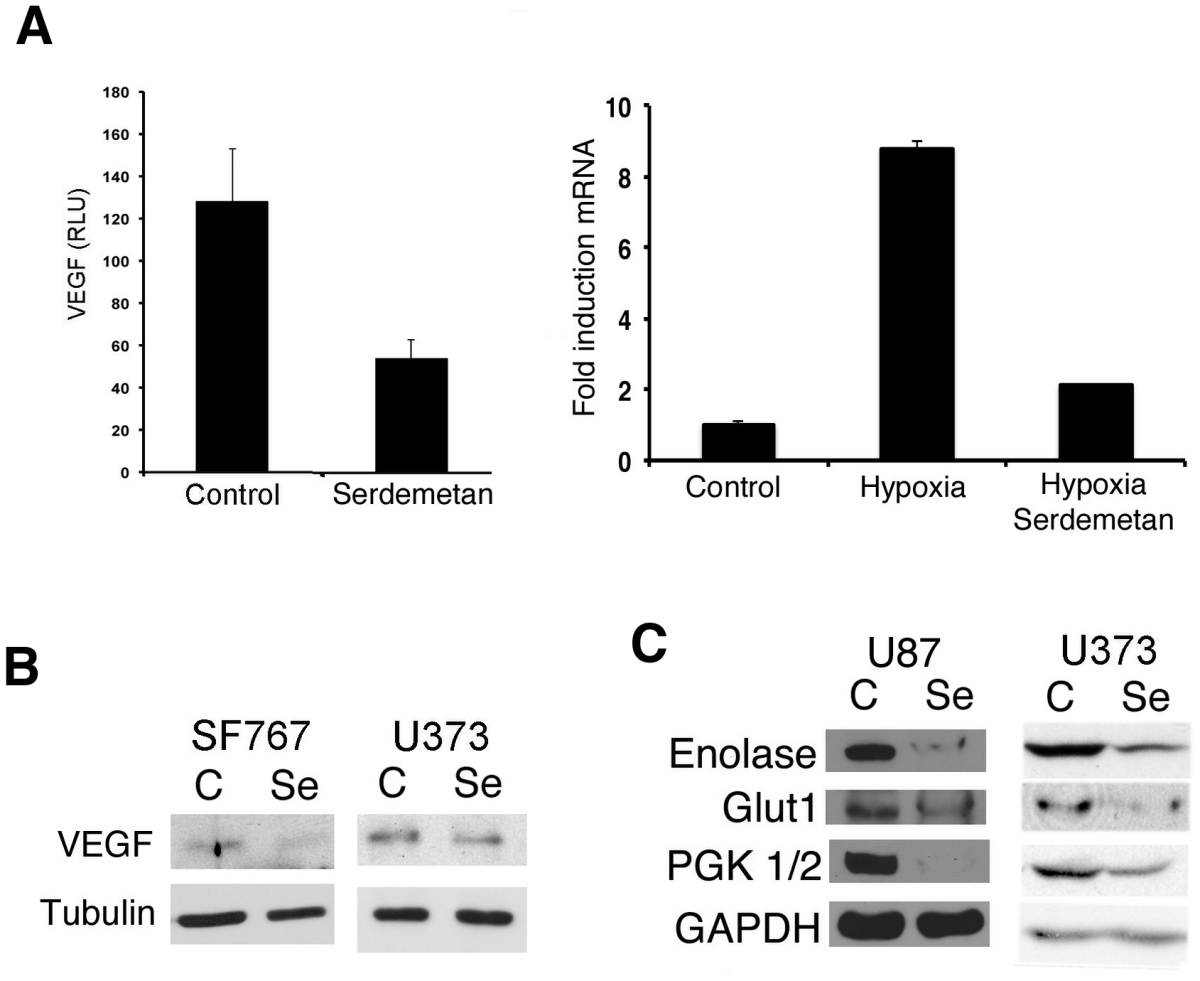
Analysis of HIF1α targets in response to Serdemetan. **A)** Luciferase assay of U87 cells transfected with the 4X HRE luciferase construct (left panel) or real time PCR for *vegf* from U87 cells (right panel) treated with 10 µM Serdemetan or DMSO under hypoxic conditions. **B)** Western blot of VEGF levels in SF767 and U373 cells. Cells were treated with 10 µM Serdemetan or DMSO control under hypoxic conditions. **C)** Western blot analysis of enolase, Glut1, PGK1/2 and GAPDH from U87 and U373 cells. Cells were treated with 30 µM of Serdemetan and subjected to hypoxia for 24 h.

HIF1α is a transcriptional factor for many genes including *vegf* and several genes involved in glycolysis. VEGF is a critical component and necessary for re-direction of blood flow and nutrients to tumor sites under hypoxic conditions. Since treatment of Serdemetan resulted in low levels of nuclear HIF1α (Φιγ. 2Β), we examined *vegf* promoter activity by luciferase reporter assay in U87 cells, and VEGF levels by western blot in U373 and SF767 cells. VEGF levels as measured by luciferase, real time PCR, and western blot were diminished in the presence of Serdemetan (Fig. 3A,and B).

**Figure 4:**
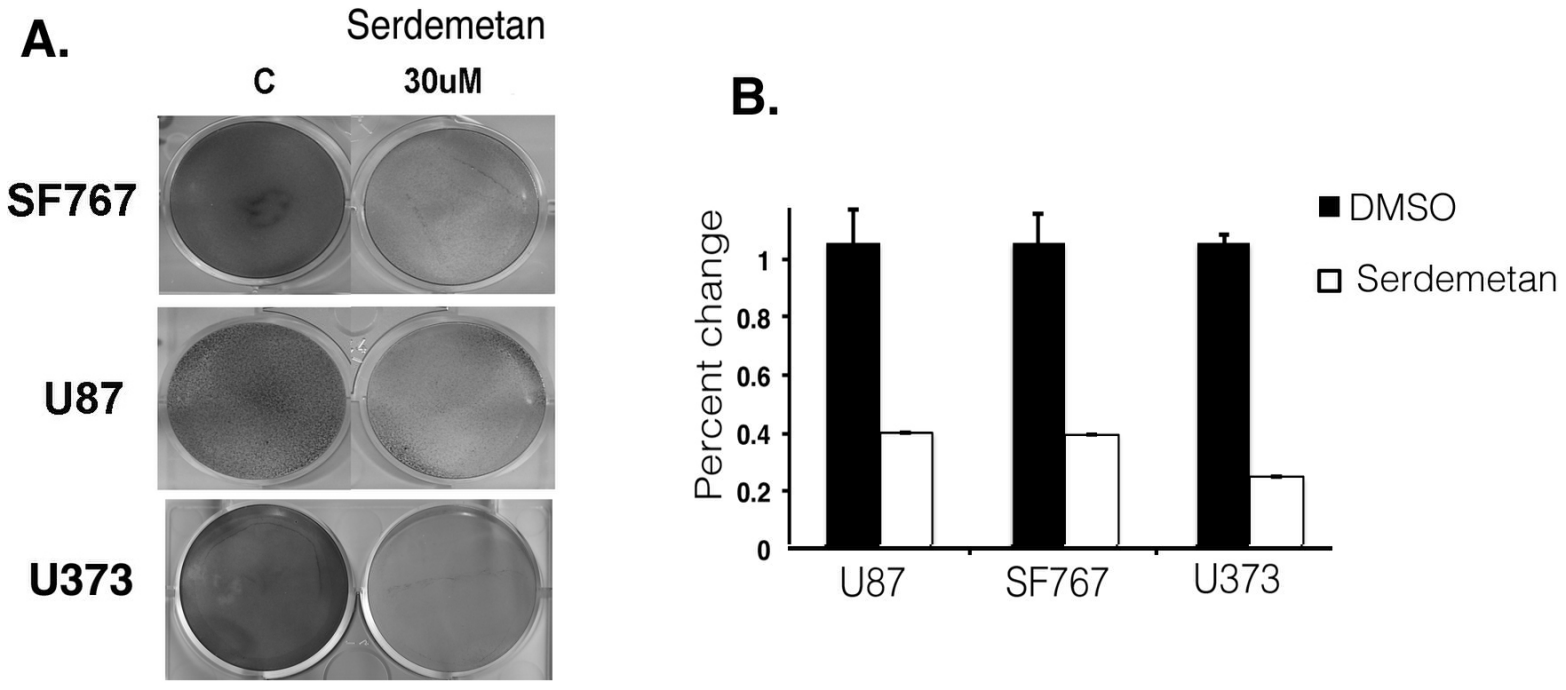
Survival of cells treated with Serdemetan. **A)** Colony forming assay was performed on SF767, U373, and U87 cells under hypoxia for 48 h with DMSO or Serdemetan. **B)** Quantitation of the colony-forming assay by measuring the absorbance at 595 nm as percent change from DMSO. Error bars represent standard deviation as calculated from the mean (n = 3).

We next examined if the expression of a select number of HIF1α glycolytic-targets were altered by Serdemetan in U87, SF767, and U373 cells under hypoxic conditions. Enolase, Glut1, MMP2 and PGK1/2 were decreased with Serdemetan treatment compared to DMSO controls (Fig. 3C). Considering that most cancer cells are addicted to glycolysis to generate energy for survival, we examined if Serdemetan would affect cell survival incubated under hypoxic conditions. We observed that treatment with Serdemetan and hypoxia resulted in a fewer number of viable cells by 48 h in a colony-forming assay (Fig. 4). This effect was independent of the p53 status as all cell lines tested were sensitive to Serdemetan.

## Discussion

Glioblastoma multiforme is characterized as a high-grade multicellular glioma subtype that represents one of the most aggressive forms of cancer with poor clinical prognosis and outcome [11]. Since human malignant glioblastoma are refractory to conventional therapeutic approaches, we examined if Serdemetan had an effect in human glioblastoma cell lines. Serdemetan has been reported to increase p53 protein levels in multiple cancer cell lines and mice [6], [7], [12]. In agreement with other studies using different cell lines, we found elevated p53 levels in U87 glioblastoma cells after Serdemetan exposure (Fig. 1A and B). While Serdemetan treatment has resulted in an increase in some p53 targets, this was not consistent in every cell line examined. Thus, it seems that this compound is not a strong activator of p53, which is evident in our study. Indeed, we observed that treatment of Serdemetan was not effective at inducing p21 in glioblastoma cells that maintain wild type p53 or gain of function mutant p53 (Fig. 1). We did observe that Serdemetan treatment modestly elevated Mdm2 protein levels, which did not depend on p53. These data suggest that engagement of p53 transcriptional activity is not the primary mechanism whereby Serdemetan may function (Fig. 1).

Rapid proliferation of tumor cells away from the vasculature results in a microenvironment of limited oxygen (1% oxygen or less). In this hypoxic environment, the transcription factor HIF1α is elevated, which induces *vegf* gene expression, a growth factor necessary for stimulating angiogenesis and permeabilization of the vessels. Detectable HIF1α protein is associated with tumor grade and vascularization of glioblastoma patients whose survival is less than a year [13]. Considering that solid tumor growth is under hypoxic conditions, we examined the responsiveness of HIF1α protein to Serdemetan exposure. We found that HIF1α and VEGF levels were lower with Serdemetan treatment (Fig. 3A and B). This observation supports the existence of an Mdm2-HIF1α-VEGF axis. This data is also congruent with our previous report showing that either loss of Mdm2 by genetic manipulation, or using pharmacological blockade to prevent Mdm2-HIF1α binding, attenuated VEGF induction (4). A recent report has demonstrated the importance of dual inhibition of Mdm2 and VEGF in neuroblastomas, which subsequently led to slower tumor growth and less vascularization of the tumors [14]. Serdemetan does not appear to alter endothelial cells as measured by *in vitro* neo-vessel formation in matrigel [7]. Collectively, Serdemetan is effective at regulating the production of VEGF by the tumor cells and not the action of VEGF on endothelial cells (Fig. 3).

It is necessary for tumor cells that have proliferated away from vessels to utilize a non-mitochondrial energy source such as glycolysis. Glycolytic genes are induced in response to limited oxygen by HIF1α. Our data show that Serdemetan led to reduced protein levels of multiple HIF1α stimulated gene targets (Fig. 3B). Not surprisingly, we found that treatment with Serdemetan was effective in decreasing cell survival of p53 wild type and p53 inactive cells (Fig. 4A and B). It was evident after 48 h that p53 cell lines were more sensitive to Serdemetan, which may relate to the fact that p53 could induce anti-metabolic pathways [15].

A recent Phase I clinical trial report of Serdemetan in patients with advanced solid tumors determined that the maximum tolerated dose of Serdemetan was 350 mg/once daily [12]. However, prolonged cardiac QT was associated with Serdemetan treatment, which led to termination of the clinical trial. This development was not surprising considering that cardiac QT can be affected by glucose metabolism, and Serdemetan treatment decreases at least several enzymes in the glycolytic pathway (Fig. 3). In summary, our current studies highlight the molecular pitfalls of using Serdemetan or other Mdm2 targeting compounds in solid tumor treatment. The fundamental Mdm2-HIF1α axis that is necessary to regulate downstream glycolytic enzymes is pivotal for normal physiological metabolism. Overall, Mdm2 governs many pathways independently of p53 and these pathways must be considered to alleviate detrimental long-term side affects in patients.
